# Nutraceutical Prevention of Diabetic Complications—Focus on Dicarbonyl and Oxidative Stress

**DOI:** 10.3390/cimb44090297

**Published:** 2022-09-18

**Authors:** Mark F. McCarty, James J. DiNicolantonio, James H. O’Keefe

**Affiliations:** 1 Catalytic Longevity Foundation, San Diego, CA 92109, USA; 2 Saint Luke’s Mid America Heart Institute, St. Louis, MO 64111, USA

**Keywords:** diabetes complications, dicarbonyl stress, oxidative stress, glyoxalase I, mitochondrial biogenesis, NADPH oxidase, glutathione, thiamine deficiency, nutraceuticals, functional foods

## Abstract

Oxidative and dicarbonyl stress, driven by excess accumulation of glycolytic intermediates in cells that are highly permeable to glucose in the absence of effective insulin activity, appear to be the chief mediators of the complications of diabetes. The most pathogenically significant dicarbonyl stress reflects spontaneous dephosphorylation of glycolytic triose phosphates, giving rise to highly reactive methylglyoxal. This compound can be converted to harmless lactate by the sequential activity of glyoxalase I and II, employing glutathione as a catalyst. The transcription of glyoxalase I, rate-limiting for this process, is promoted by Nrf2, which can be activated by nutraceutical phase 2 inducers such as lipoic acid and sulforaphane. In cells exposed to hyperglycemia, glycine somehow up-regulates Nrf2 activity. Zinc can likewise promote glyoxalase I transcription, via activation of the metal-responsive transcription factor (MTF) that binds to the glyoxalase promoter. Induction of glyoxalase I and metallothionein may explain the protective impact of zinc in rodent models of diabetic complications. With respect to the contribution of oxidative stress to diabetic complications, promoters of mitophagy and mitochondrial biogenesis, UCP2 inducers, inhibitors of NAPDH oxidase, recouplers of eNOS, glutathione precursors, membrane oxidant scavengers, Nrf2 activators, and correction of diabetic thiamine deficiency should help to quell this.

## 1. How Hyperglycemia Drives Diabetic Complications

The complications of diabetes reflect dysfunction or death of cell types that are highly permeable to glucose in the absence of effective insulin activity. These cells include podocytes, mesangial and tubular cells of the kidney, vascular endothelial cells, neurons, glia, and immune cells [1]. (Note that the macrovascular complications of diabetes—atherosclerosis and cardiomyopathy—appear to stem from dysfunctional vascular endothelium [2].) Such cells express glucose transporters whose activity is not dependent on insulin signaling. As a result, glycolytic intermediates build up to excess levels, and increased accumulation of pyruvate may impose a substrate load on mitochondria. In a classic model, Brownlee and colleagues have proposed that increased levels of certain glycolytic intermediates can provoke cellular damage by inducing oxidative stress (via mitochondria and PKC activation of NADPH oxidases), dicarbonyl stress, an accelerated polyol pathway, and excess O-GlcNAcylation of proteins [1,3]. Especially in type 2 diabetics, episodic elevations of free fatty acids at physiologically inappropriate times (reflecting adipocyte insulin resistance) may collaborate with hyperglycemia in the induction of diabetic complications—by boosting diacylglycerol levels [4]. It may be feasible to prevent or at least ameliorate diabetic complications by inhibiting several or all of these mechanisms. This essay examines nutraceutical strategies which may have potential in this regard—with particular emphasis on the induction of glyoxylase 1 as a technique for coping with dicarbonyl stress.

## 2. A Key Role for Dicarbonyl Stress in Diabetic Complications

Although considerable evidence points to oxidative stress as a major driver of diabetic complications, so-called “dicarbonyl stress” has also emerged as a key driver of these complications, the effects of which are mediated only in part by reactive oxygen species (ROS) [1,5]. Hence, any plan to prevent diabetic complications much include components intended to address dicarbonyl stress. In cell types exposed to hyperglycemia that are highly permeable to glucose in the absence of insulin signaling, the glycolytic triose phosphates glyceraldehyde-3-phosphate and dihydroxyacetone phosphate accumulate. These are susceptible to spontaneous loss of phosphate, giving rise to the highly reactive dicarbonyl compound methylglyoxal; the rate of this reaction is directly proportional to the concentration of triose phosphates [6]. Methylglyoxal reacts most readily with the head groups of arginine residues in proteins, giving rise to a structure known as hydroimidazolone (M-H1); this, along with the hydroimidazolone derived from 3-deoxyglucosone, is the most common advanced glycation end product (AGE) found in the plasma and tissues of diabetics [7].

M-H1, as well as arginine-containing proteins or peptides which feature it, can interact with very high (nanomolar) affinity with the receptor for advanced glycation end products—the RAGE receptor—triggering its activation [8]. This affinity is far higher than RAGE’s affinity for certain other commonly discussed lysine-linked AGEs elevated in diabetics such as N^ε^-carboxymethyl-lysine, and hence is more likely to be of physiologically significance [8]. RAGE activation promotes inflammation via stimulation of NF-kappaB activity, can boost ROS production by activating NADPH oxidase, and can drive proliferation via PI3K-Akt and ERK signaling; its role in the genesis of diabetic complications is well established [9]. RAGE activation up-regulates its own expression, as NF-kappaB drives the transcription of its gene; this makes RAGE activators that much more potent as triggers for inflammation [10]. Additionally, methylglyoxal’s protein-binding activity can trigger endoplasmic reticulum stress—another source of inflammation—by impairing protein folding [11]. This may reflect in part the ability of M-H1 to promote protein cross-linking, as it contains a carbonyl group susceptible to attack by nucleophilic cysteine groups [12]. Methylglyoxal-mediated damage to the mitochondrial electron transport chain boosts superoxide generation while impairing ATP synthesis [13,14]. Additionally, methylglyoxal can react directly with 2′-deoxyguanosine residues in DNA, promoting DNA damage and mutagenesis [15]. Administration of methylglyoxal to normoglycemic mice has been reported to produce vascular and renal damage analogous to that seen in diabetics [16,17,18].

These considerations suggest that it is hardly accidental that cells have devised a system for detoxifying methylglyoxal that has been conserved throughout evolution. The enzymes glyoxalase 1 and 2, acting in tandem with a catalytic assist from glutathione, convert methylglyoxal to harmless lactate [19,20]. In effect, this reaction converts one of the carbonyls to a carboxyl group, and the other to a hydroxyl group—there is no net oxidation or reduction, and the end product is innocuous. Glyoxylase 1 (Glo1) is rate-limiting for this process, and mice with genetic overexpression of Glo1 are protected from renal, retinal, and endothelial dysfunction and damage when rendered diabetic [1,21,22,23,24]. Conversely, normoglycemic mice with Glo1 knock-down display renal damage analogous to that seen in diabetics [25]. These findings suggest that safe, practical strategies for boosting Glo1 expression and/or activity may have important potential in diabetes management.

Attention has been drawn to mechanisms which regulate transcription of the Glo1 gene. The promoter of this gene has been found to contain response elements that are positively responsive to insulin signaling, the Nrf2 transcription factor, and the metal-responsive transcription factor 1 (MTF-1) [26,27]. The fact that insulin activity boosts Glo1 expression makes perfect sense homeostatically: insulin signals elevated glucose, and elevated glucose leads to increased methylglyoxal production in glucose-permeable tissues.

## 3. Nutraceutical Induction of Glyoxalase 1 Expression

The transcription-promoting activity of MTF-1 is activated by binding to zinc; it also mediates zinc-induced increased expression of the antioxidant protein metallothionein [28,29]. Addition of extra zinc to the culture medium of HepG2 cells transfected with the promoter of the Glo1 gene was associated with doubling of promoter activity [26]. This finding may well be pertinent to the clinical literature correlating lower plasma zinc levels in diabetics with increased risk for diabetic complications, including nephropathy, retinopathy, neuropathy, and cataracts [30,31,32,33,34,35]. These findings, however, should be viewed circumspectly inasmuch as hyperglycemia impairs renal retention of zinc; diabetics therefore tend to be relatively zinc deficient, and lower zinc status in diabetics may thus be a marker for poorer diabetic control [36,37]. The causative association between zinc status and diabetic complications is better established by studies in diabetic rodents—concurrent zinc deficient has been found to aggravate diabetic complications, whereas zinc supplementation has been found to be protective with respect to such complications [36,38,39,40,41,42,43,44,45,46,47,48,49,50,51,52,53,54,55,56,57,58]. Two small, short term controlled clinical trials of zinc supplementation observed improvements in peripheral neuropathy, as assessed by motor nerve conduction velocity [59,60].

Good zinc status has the further merit that it modestly improves insulin sensitivity—likely through reversible inhibition of tyrosine phosphatase 1B activity targeting the insulin signaling pathway [37,61,62]. Moreover, zinc-inducible metallothionein is an effective scavenger for peroxynitrite-derived radicals—which, in diabetics, promote uncoupling of endothelial nitric oxide synthase by oxidizing its cofactor tetrahydrobiopterin, thereby boosting superoxide generation and impairing the nitric oxide generation vital for vascular health [63,64,65,66,67,68]. Additionally, supplemental zinc can function as an antagonist of the toxicity of cadmium, which has been linked a range of adverse health outcomes even at ambient non-industrial exposure levels [69]. Remarkably, supplementation with 80 mg zinc daily (accompanied by 2 mg copper to prevent induction of copper deficiency) was associated with a highly significant 27% reduction in total mortality over 6 years of follow-up (RR, 0.73; 95% CI, 0.61–0.89) in the AREDS1 study examining the impact of nutritional supplements on progression of early age-related macular degeneration in the elderly [70]. In light of these findings, a clinical trial examining the impact of graded doses of supplemental zinc on tissue expression of Glo1 and plasma levels of M-H1 in diabetics is clearly warranted. More ambitiously, a long-term randomized controlled trial evaluating the impact of ample zinc supplementation on the development of complications in diabetics would be appropriate—particularly in light of the provocative mortality findings in the AREDS1 trial, that have been mostly ignored.

Nutraceuticals clinically useful for Nrf2 activation—so-called phase 2 inducers—also have potential as Glo1 inducers [27]. Some of these work by interacting covalently cysteinyl residues of Keap1, preventing it from binding Nrf2 in the cytoplasm, and thereby enabling Nrf2 to be transported to the nucleus where it can promote transcription not only of Glo1, but also an entire panoply of antioxidant enzymes (including the enzyme rate-limiting for glutathione synthesis, γ-glutamylcysteine transferase) that could be expected to protect diabetic tissues from the adverse effects of excess ROS production [71,72,73,74]. Isothiocyanates (such as sulforaphane) derived from lightly cooked cruciferous vegetables can act as Keap1-binding Nrf2 activators [71,75]. Lipoic acid, in its oxidized form, likewise binds Keap1 and activates Nrf2 activity; it has been explored as an agent for treating diabetic neuropathy [76,77,78,79]. The endogenous gasotransmitter hydrogen sulfide also binds to Keap1 and activates Nrf2—an effect which may contribute notably to the health protection associated with adequate H_2_S production [80,81]. Supplemental taurine can boost the expression of enzymes catalyzing H_2_S production in vascular tissues—an effect which may explain taurine’s favorable impact on vascular health—and supplemental N-acetylcysteine can increase the availability of cysteine, the key precursor for H_2_S generation [82,83,84,85,86].

Other nutraceuticals can boost Nrf2 synthesis; melatonin does so by stimulating the clock transcription factor Bmal1, which binds to the promoter of the Nrf2 gene and drives its transcription [87,88,89,90]. Another nutraceutical with the potential to promote transcription of this gene is astaxanthin; this can serve as an agonist for the aryl hydrocarbon receptor, which, like Bmal1, can bind the Nrf2 promoter and drive its transcription [91,92,93,94,95,96]. Hence, the antioxidant benefits of astaxanthin extend far beyond its ability to serve as a highly efficient scavenging antioxidant for biological membranes—most notably the mitochondrial inner membrane [97,98,99].

Although the amino acid glycine is not a direct activator of Nrf2, a recent study shows that, in the context of hyperglycemia (but not normoglycemia), exposure to increased levels of glycine promotes migration of Nrf2 to the nucleus, much like Nrf2 activators do [100]. The mechanistic basis of this effect currently remains obscure. It is intriguing that methylglyoxal itself can promote nuclear uptake of Nrf2, by inducing a crosslinking of Keap1 subunits that inhibits their ability to interact with Nrf2; could glycine somehow potentiate this cross-linking [12]? In any case, oral glycine supplementation has been shown to boost expression of Glo1 and down-regulate RAGE signaling in the aorta of diabetic rats [101]. Moreover, supplemental glycine may boost the activity of pre-existing Glo1 by increasing glutathione levels [102]; although cysteine availability (which can be amplified with supplemental N-acetylcysteine) is generally considered rate-limiting for glutathione synthesis, glycine availability also has a regulatory impact in this regard, and joint supplementation with N-acetylcysteine and glycine has shown profound antioxidant effects in both rodent and clinical studies [103,104,105,106,107,108]. Indeed, such supplementation has been reported to reduce insulin resistance in type 2 diabetics, while decreasing their plasma methylglyoxal levels (this latter effect presumably reflecting enhancement of Glo1 activity by elevated glutathione) [107]. Rodent studies reporting that supplemental glycine can prevent cataracts in diabetic rats may conceivably reflect increased Glo1 induction [109,110]. Glycine supplementation also exerts anti-inflammatory effects by activation of glycine receptors expressed on the plasma membranes of many types of myeloid cells [111,112]. The use of high-dose glycine in diabetes management is rendered practical by the fact that glycine is highly soluble, has a pleasant mildly sweet flavor, and is quite inexpensive—for example, it can be employed as a sweetener in coffee or tea [102].

In aggregate, these considerations suggest that the contribution of dicarbonyl stress to diabetic complications may be addressable with a nutraceutical regimen that incorporates zinc, glycine, and one or more agents that target nrf2 activation—such as lipoic acid, sulforaphane (as from broccoli sprout extracts), melatonin and astaxanthin. Fortuitously, each of these agents also can aid antioxidant defenses. More generally, combining a regimen for Glo1 induction with a comprehensive antioxidant supplementation program, targeting the sources of oxidative stress activated in diabetics, may have substantial practical potential for prevention of diabetic complications, provided that complex nutraceutical supplements and functional foods are developed that make such a program fairly easy for patients to implement.

## 4. Sources of Diabetic Oxidative Stress

The oxidant stress evoked by hyperglycemia, as amplified by the free fatty acid excess associated with insulin resistance, can adversely alter cellular function via such mechanisms as up-regulation of MAP kinase and NF-kappaB signaling, support of transforming growth factor-β pro-fibrotic activity, uncoupling of endothelial nitric oxide synthase, and induction of DNA damage with PARP activation [113,114,115,116,117,118,119,120]. The key sources of oxidant stress in diabetes appear to be structurally damaged mitochondria processing excess substrate, activated NADPH oxidase complexes (particularly NOX2 and NOX4), and uncoupled endothelial nitric oxide synthase (eNOS) [1,5].

## 5. Nutraceuticals for Promoting Mitophagy and Mitochondrial Biogenesis

Methylglyoxal-mediated damage to the mitochondrial electron transport chain, in conjunction with increased oxidizable substrate provided by enhanced glycolysis and increased free fatty acids, can boost oxidant production by mitochondria. Measures which promote mitophagy of damaged mitochondria, while boosting compensatory mitochondrial mitogenesis, could be expected to quell excessive mitochondrial ROS generation. A recent essay has addressed nutraceuticals with potential for promoting this complex process [121]—they include agents which boost Sirt1 activity (see below), activate AMPK (berberine—a nutraceutical derived from the rhizomes of *Coptis chinensis*, used in traditional Chinese medicine for diabetes control, and now well documented to aid glycemic control in type 2 diabetics), stimulate Nrf2 (as discussed above), and activate PPARα (astaxanthin), as well as the dietary polyamine spermidine [122,123,124,125,126,127,128,129,130,131]. In addition, astaxanthin can act as a highly efficient oxidant scavenger for the mitochondrial inner membrane [98,132,133].

With respect to Sirt1 activation—crucial for efficient mitophagy as well as mitochondrial biogenesis—resveratrol, which can boost Sirt1 activity via allosteric interaction, has been somewhat disappointing clinically owing to its inefficient absorption and rapid metabolism; nonetheless, modest reductions in systolic blood pressure and hemoglobin A1c have been observed when diabetics have been treated with resveratrol [134,135,136,137,138]. Nutraceuticals with greater clinical potential for Sirt1 activation include several that somehow enhance Sirt1 synthesis—such as ferulic acid [139,140,141,142], melatonin [143,144,145], tetrahydrocurcumin [146,147], and urolithin A [148,149,150]—and N1-methylnicotinamide, a natural nicotinamide metabolite that increases the half-life of the Sirt1 protein [151]. (Ferulic acid, tetrahydrocurcumin, and urolithin A are major circulating metabolites of ingested anthocyanins, curcumin, and pomegranate ellagitannins, respectively, thought likely to mediate the health benefits of these compounds.) Cellular levels of Sirt1′s obligate cofactor NAD+ can be increased with nicotinamide riboside or nicotinamide ribonucleotide, both available as nutraceuticals [152,153,154,155,156,157]. Additionally, berberine-mediated activation of AMPK stimulates rapid reconversion of nicotinamide to NAD+ by promoting induction of nicotinamide phosphoribosyltransferase [158,159].

The ability of thymoquinone (a key component of *Nigella sativa*—black cumin seed—oil) and of pyrroloquinoline quinone (PQQ)—a vitamin-like compound in the diet that binds with high affinity to lactate dehydrogenase—to boost Sirt1 activity rests in their ability to promote oxidation of NADH to NAD+. The antioxidant enzyme NAD(P)H quinone oxidoreductase 1 (NQO1) can reduce thymoquinone to thymohydroquinone, converting NADH to NAD+ in the process; thymohydroquinone can then act as a scavenging antioxidant [160]. This mechanism can explain thymoquinone’s ability to activated Sirt1 [161,162,163]. NQO1 has been found to bind to Sirt1, and hence, in the presence of reducible quinones, functions physiologically to furnish Sirt1 with the NAD+ it requires [164,165]. In a reaction catalyzed by lactate dehydrogenase, PQQ is reduced and NADH converted to NAD+ in the process [166]. This makes lactate more available for oxidation—while also generating NAD+ needed for Sirt1 activity [167,168]. Thymoquinone can also function as a Keap1-binding Nrf2 activator, giving it particular utility as an antioxidant [169,170,171]. Thymoquinone can be provided by capsules of *Nigella sativa* oil (standardized to 2–3% potency), and PQQ is available as a pure chemical.

It should be noted that Sirt1 activation has been found to suppress the range of diabetic complications—nephropathy, retinopathy, neuropathy, cardiomyopathy, cataract—in rodent models of diabetes [172,173]. Sirt1 can deacetylate and thereby modulate a wide range of proteins, and these protective effects may stem from mechanisms that are not fully dependent on improved mitochondrial biogenesis. In particular, Sirt1 opposes the pro-inflammatory activity of NF-kappaB via deacetylation of p65 [174].

## 6. Boosting Expression of Mitochondrial Uncoupling Proteins

The propensity of hyperglycemia to boost mitochondrial oxidant production in glucose-permeable cells can be decreased by increased expression of UCP family uncoupling proteins; by enabling protons to flow back into the mitochondrial matrix, these proteins moderate the elevated mitochondrial electrochemical potential induced by high Krebs cycle activity that results in increased mitochondrial superoxide generation [175,176]. PPARalpha agonists—such as astaxanthin—can increase expression of these proteins [177]. PPARdelta exerts a similar effect and, in certain cell types, such as vascular endothelium, capsaicin-mediated activation of the transient receptor potential vanilloid 1 (TRPV1) receptor boosts its expression and activity [178,179,180]. In endothelial cells exposed to hyperglycemia, capsaicin exposure enhances UCP2 expression and markedly reduces ROS production [180,181]. In diabetic mice, capsaicin administration likewise alleviated diabetes-induced endothelial dysfunction—an effect negated by UCP2 knockout [180]. The extent to which these effects can be generalized to other glucose-permeable tissues involved in diabetes complications remains to be assessed. Favorable effects of capsaicin feeding on nephropathy and cardiomyopathy in diabetic rodents have been reported [181,182]. Capsaicin, a potent agonist for TRPV1, is the compound responsible for the “heat” of chili peppers, and there is growing evidence that it has important health-protective potential [183]. Prospective Chinese epidemiology has linked regular chili pepper consumption to decreased risk for weight gain—an effect which might be expected with an uncoupling protein inducer [184]. High chili consumption has also been linked to markedly lower cardiovascular, cancer and global mortality, as established by a recent meta-analysis (RR = 0.75 [95% CI: 0.64–0.88; *p* = 0.0004] for all-cause mortality). For those who do not enjoy the culinary excitement imparted by hot chili, capsaicin supplements featuring cayenne pepper are available for nutraceutical use.

## 7. Controlling NADPH Oxidase Activity

Increased levels of the glycolytic intermediate glyceraldehyde-3-phosphate, after reduction to glycerol-3-phosphate, can lead to de novo generation of diacylglycerol, particularly in the context of elevated free fatty acids; diacylglycerol, via activation of protein kinase C, can promote assembly and activation of NOX2-dependent NAPDH oxidase; this mechanism is thought to be largely responsible for elevated NADPH oxidase activity in diabetes [4,185,186,187]. Diabetes can also promote increased expression of NOX4 [188,189]. The free bilirubin generated by induction of heme oxygenase activity can function physiologically as an inhibitor of certain NADPH oxidase complexes, including NOX2 and NOX4 [190,191,192,193,194]. Diabetics with chronically elevated plasma free bilirubin levels owing to Gilbert syndrome are markedly protected from diabetic complications, independent of serum glucose level [195]. Although strategies for boosting plasma levels of free bilirubin have been proposed for management of diabetes and other NADPH oxidase-linked disorders, a nutraceutical strategy may prove to be more practical [196,197]. Phycocyanobilin (PCB), a biliverdin derivative that functions as a light-absorbing chromophore found in cyanobacteria (such as the food spirulina) and certain blue-green algae, appears to mimic the NADPH oxidase-inhibitory impact of bilirubin—a fact which may explain, in part, the potent antioxidant and anti-inflammatory activities of orally administered spirulina or phycocyanin (the spirulina protein to which PCB is covalently attached) in a wide range of rodent models of disease [197,198,199,200]. Oral administration of either phycocyanin or PCB has been shown to protect diabetic db/db mice from diabetic nephropathy [198]. Hence, adequate intakes of spirulina (or of more concentrated sources of phycocyanin or PCB) may have important antioxidant potential in diabetics.

## 8. Recoupling eNOS and Mimicking Its Benefits

In the context of diabetes, eNOS tends to become uncoupled, both because peroxynitrite-derived radicals can oxidize its essential cofactor tetrahydrobiopterin to dihydrobiopterin, and oxidant-mediated inactivation of dimethylarginine dimethylamino-hydrolase (DDAH) increases cellular levels of asymmetric dimethylarginine (ADMA), a functional competitor of arginine’s association with eNOS [66,119,201,202,203,204]. These effects are doubly pernicious, as they turn eNOS into a prolific source of superoxide while impeding its ability to produce vascular-protective low-dose nitric oxide. Supplementation with high-dose folate and with the amino acid citrulline can reverse this uncoupling [205,206]. High-dose folate, via induction of increased expression of dihydrofolate reductase, promotes reduction of dihydrobiopterin back to its active tetrahydrobiopterin form [207,208]. Citrulline—more efficiently absorbed and transported to tissues than arginine—is readily converted to arginine within cells, thereby opposing the adverse effect of elevated ADMA on eNOS activity [206,209,210].

The bioactivity of eNOS-generated NO is impaired not only by eNOS uncoupling, but also by a direct quenching of NO by superoxide, yielding the unstable oxidant peroxynitrite. NO-mediated stimulation of soluble guanylate cyclase (sGC) and consequent production of cyclic GMP (cGMP) plays an important role in the prevention of diabetic nephropathy, neuropathy, cardiomyopathy and the endothelial dysfunction promoting atherosclerosis, as can be judged by the fact that treatment with drugs that directly stimulate sGC or that inhibit phosphodiesterase-5 (PDE-5, which selectively degrades cGMP) is protective with respect to these complications in rodent diabetes models [211,212,213,214,215,216,217,218,219,220,221,222,223,224,225,226]. High doses of the B vitamin biotin likewise have potential in this regard, as, in pharmaceutically feasible concentrations about a hundred-fold higher than the physiological plasma level, biotin can serve as an agonist for sGC [227,228,229,230,231,232]. Hence, despite the absence of any published animal studies assessing the impact of high-dose biotin on diabetic complications, there is reason to suspect that biotin could be clinically worthwhile for this purpose. A small case series suggests that 4–8 weeks of supplemental biotin (5–10 mg daily) can achieve marked improvements of clinical and laboratory findings in diabetic neuropathy [233]. Although high-dose biotin is well tolerated—its stimulatory impact on sGC is modest, and hence unlikely to precipitate hypotension—it has the drawback that it can interfere with certain lab assays that employed biotinylated substrates; hence, it is prudent to discontinue its use for at least several days prior to important lab tests [234,235].

Xanthine oxidase activity is expressed in certain diabetic tissues, notably the kidney, and can contribute to superoxide generation; however, research attention has focused on its product uric acid as a possible mediator of diabetic complications [236]. Allopurinol and certain other pharmaceutical xanthine oxidase inhibitors have a protective impact on diabetic nephropathy in rodents, but allopurinol has failed to benefit diabetic renal function in lengthy clinical trials [237,238,239]. Conceivably, the benefit seen in rodent studies reflects the fact that xanthine oxidase product uric acid can boost NADPH oxidase activity in some tissues [240,241,242]. However, this effect is maximized at levels below normal human plasma levels—whereas normal plasma levels in rodents are far lower owing to their expression of uricase. Curiously, an increase of uric acid in humans can actually have a net antioxidant effect owing to its ability to scavenge peroxynitrite-derived radicals [243,244]. Meta-analyses of clinical trials also find that allopurinol fails to improve endothelial function or glycemic control in diabetics [245,246]. Targeting xanthine oxidase does not appear to have much promise for quelling complications of diabetes in humans.

## 9. Inducing Nrf2 and Correcting Thiamine Deficiency

Nutraceuticals which enhance Nrf2 activity—as discussed above—could be expected to counteract the adverse consequences of superoxide/hydrogen peroxide generation in the tissues of diabetics. As noted, zinc-mediated induction of metallothionein could be protective in this regard as well. Additionally, supplemental glycine and N-acetylcysteine can collaborate in enhancing tissue glutathione levels [102].

One key effect of Nrf2 inducers is to increase expression of glucose-6-phosphate dehydrogenase (G6PD) and 6-phosphogluconate dehydrogenase (6PGD)—enzymes in the upper pentose phosphate pathway that generate the NADPH reducing power required for glutathione and thioredoxin to act effectively as antioxidants [247,248]. However, diabetics are prone to sub-optimal thiamine status, as hyperglycemia suppresses the ability of the renal proximal tubules to reabsorb thiamine [249]. Poor thiamine status compromises the activity of the enzyme transketolase, an essential mediator in the lower pentose phosphate pathway; this in turn leads to intracellular build up of certain products of the pentose phosphate pathway that can allosterically inhibit both G6PD and 6PGD, impeding NADPH generation [250,251]. Hence, correcting poor thiamine status in diabetics with high-dose thiamine supplementation, or with high-absorption thiamine precursors such as benfotiamine or dibenzoylthiamine, can exert a worthwhile antioxidant effect in diabetics [252,253].

## 10. Other Suspected Mechanisms for Diabetic Complications—The Glucosamine and Polyol Pathways

A potential mechanism for promotion of diabetic complications, independent of either oxidative stress or dicarbonyl stress, is increased O-GlcNAcylation, reflecting greater availability of the fructose-6-phosphate that is the first substrate for generation of UDP-N-acetylglucosamine [254]. Although increased O-GlcNAcylation can compromise insulin sensitivity via effects on mediators of insulin signaling, this is clearly not the primary mechanism for insulin resistance in type 2 diabetics or hyperglycemia in type 1 diabetics [255]. While there has been considerable speculation that O-GlcNAcylation is a key mediator of diabetic complications, the evidence on this point is not yet clear, reflecting lack of a drug that can be employed quite specifically to inhibit O-GlcNAc transferase activity. Moreover, recent epidemiology finds that regular glucosamine supplementation—which would be expected to enhance cellular pools of UDP-N-acetylglucosamine, and hence up-regulate O-GlcNAcylation—is associated with a marked reduction in total mortality, including cardiovascular mortality [256,257,258,259]. Increased longevity has also been reported in mice supplemented with glucosamine beginning in middle age [260]. Glucosamine supplemented at 3 g daily in humans has been found to enhance flow-mediated vasodilation—a marker for endothelial health [261]. Favorable effects of glucosamine on longevity might reflect the fact that O-GlcNAcylation of Sirt1—an enzyme with important anti-inflammatory, antioxidant, and pro-autophagic effects that promote increased healthspan [262]—enhances its catalytic activity [263]. Hence, suppressing glucosamine generation in diabetics, or down-regulating O-GlcNAc transferase activity—if and when a safe drug or nutraceutical capable of doing this is identified—would likely be a mixed blessing at best, and might not prove to be an effective remedy for diabetic complications.

The enzyme aldose reductase is expressed in some tissue susceptible to damage in diabetes and uses NADPH to reduce glucose to sorbitol. This in turn can be reoxidized to fructose by sorbitol dehydrogenase, in a reaction that reduces NAD+ to NADH [264]. The net impact of this polyol pathway is to diminish the pool of NADPH required for the antioxidant efficacy of glutathione and thioredoxin, while also decreasing the pool of NAD+ required for Sirt1 activity. As noted, Sirt1 plays a key role in promoting mitochondrial biogenesis, while also dampening inflammation by opposing the transcriptional activity of NF-kappaB [174].

Drug inhibitors of aldose reductase such as sorbinil have been tested in rodent models of diabetes, and are also receiving clinical evaluation [264,265]. While phytochemicals are suggested to have potential for inhibiting aldose reductase, it is not clear that any such compounds now available in supplement form are effective for this purpose in clinically feasible concentrations [266,267,268]. Hence, the most effective current way that nutraceuticals might be employed to counteract the adverse impact of the polyol pathway is to compensate for its adverse impact on the NADPH and NAD+ pools. Nrf2 activators increase the expression of the rate-limiting enzymes in the upper pentose phosphate pathway responsible for regenerating NADPH from NADP+. Additionally, as we have noted, restoring good thiamine status in diabetics, by normalizing transketolase activity, alleviates the allosteric inhibition of these rate-limiting enzymes. With respect to NAD+, both thymoquinone and PQQ can promote oxidation of NADH to NAD+. Hence, they may be expected to counteract the adverse impact of the polyol pathway on Sirt11 activity.

In tissues with high glucose permeability in which sorbitol dehydrogenase activity is low relative to aldose reductase activity, the osmotic stress induced by sorbitol accumulation within cells has the potential to induce cell dysfunction. This phenomenon may play a role in the induction of diabetic cataracts [269]. However, rodent studies with sorbitol dehydrogenase inhibitors suggest that osmotic imbalance is not a major cause of neuronal or vascular dysfunction in diabetes [270]. Curiously, control of oxidative stress limits the ability of osmotic stress to induce cataracts in rodents, suggesting a complementary interaction of these two types of stress in cataract induction [269].

## 11. Toward a Practical Nutraceutical Strategy for Prevention of Diabetic Complications

Hence, considering our current understanding, measures which counteract oxidative stress and/or dicarbonyl stress appear to have the greatest promise for prevention of diabetic complications—in conjunction with measures that can improve glycemic control, of course. Zinc, glycine, and the range of clinically active nutraceuticals which boost Nrf2 activation (lipoic acid, broccoli sprouts, taurine, N-acetylcysteine) may be useful for alleviating dicarbonyl stress. In addition to these agents, promoters of mitophagy and mitochondrial biogenesis (such as ferulic acid, melatonin, urolithin A, N1-methylnicotinamide, nicotinamide riboside, thymoquinone, PQQ, berberine, astaxanthin, spermidine), UCP2 inducers (astaxanthin, capsaicin), inhibitors of NAPDH oxidase (spirulina), recouplers of eNOS (high-dose folate, citrulline), and correction of diabetic thiamine deficiency should help to quell the oxidative stress associated with diabetes. A survey of the pertinent biomedical literature—as by searching for the agent along with key word “diabetes” on pubmed.gov—will readily reveal that each of these agents, apart from spermidine, has been found to alleviate diabetic complications in rodent studies. (Spermidine is still little researched as a supplement but appears to have remarkable health protective potential [130,271,272,273,274,275,276]). Table 1 summarizes this proposal.

While this prescription might appear to be impossibly complicated, it should be feasible to devise a supplementation program comprising functional foods and several capsule or tablet supplements which would make it reasonably practical to implement. Table 2 provides a provisional sketch of how this might be achieved. The authors do not mean to imply that the suggested program is clinically proven or ideal in its composition; the possibility of unforeseen countervailing effects should be borne in mind. The doses chosen are within ranges that might be expected to have some physiological impact in light of previous clinical studies. The suggested program is not patented, and may be replicated in whole or in part by any nutraceutical manufacturer who cares to do so. Importantly, the agents involved are inexpensive compared to many prescription drugs and can reasonably be presumed to be safe in the suggested doses. Moreover, they might benefit health in several additional ways.

## 12. Some Agents Reducing Risk for Complications May Also Aid Insulin Function

In particular, there is evidence that some of these agents may improve muscle or adipocyte insulin sensitivity and glycemic control in type 2 diabetics or those with metabolic syndrome. Mitochondrial mass and oxidative capacity in skeletal muscle tends to be low in patients with these disorders, and studies suggest that people at increased genetic risk for diabetes tend to be deficient in this respect [277,278]. In the context of fatty diets and/or metabolic syndrome, impaired muscle capacity to oxidize fatty acids can lead to an accumulation of fatty acid derivatives in skeletal muscle—notably, diacylglycerol or ceramide—that, via activation of novel forms of protein kinase C and subsequent downstream activation of the kinases JNK and IKKβ, results in phosphorylations of insulin-responsive substrate-1 (IRS-1) that impede insulin signaling [279,280,281,282]. Concurrently, ceramide acts via atypical PKC-ξ and protein phosphatase 2A to suppress Akt activity [283,284,285]. Hence, measures which boost mitochondrial biogenesis in muscle—including nutraceuticals and exercise training—have the potential to alleviate muscle insulin resistance [286,287]. Astaxanthin can promote insulin sensitivity in diabetics and pre-diabetics in an additional way—by boosting adiponectin production [128,288,289,290,291,292]. Acting as an agonist for PPARα, astaxanthin can promote hepatic secretion of fibroblast growth factor 21 (FGF21), which in turn acts on adipocytes to increase their secretion of adiponectin [293,294,295]. The latter, via its characteristic receptor on skeletal muscle, activates a ceramidase activity that promotes insulin sensitivity by decreasing elevated ceramide levels [296,297].

Insulin resistance of the hypertrophied adipocytes in metabolic syndrome appears to be driven by increased oxidant production stemming from both NADPH oxidase (Nox2, Nox4) and mitochondria [298,299,300,301]. Infiltrating macrophages attracted by chemotactic factors also contribute to this oxidant production; moreover, oxidants up-regulate macrophage secretion of tumor necrosis factor-a (TNFα) [302]. This oxidant stress in conjunction with TNFα activity promotes JNK and IKKβ activation in adipocytes, which in turn induces adipocyte insulin resistance via IRS-1 phosphorylation—a pathway homologous to that seen in insulin resistant skeletal muscle [301,303]. Hence, suppression of NADPH oxidase activity with PCB or whole spirulina has the potential to alleviate adipocyte insulin resistance [299]. This rationalizes the insulin sensitizing effects observed when rodent or human type 2 diabetics are fed spirulina [304,305]. It is also reasonable to expect that the downstream impact of oxidants on JNK/IKKβ might be blunted to some degree by Nrf2-inducible enzymes; consistent with this possibility, lipoic acid supplementation has been reported to achieve modest dose-dependent improvements in glycemic control in type 2 diabetics [306].

The ability of berberine—an AMPK activator used commonly as an alternative to metformin in China—and of supplemental zinc to improve glycemic control in diabetics has been noted above [68,125,307]. A limited amount of clinical and pre-clinical literature suggests that high-dose biotin may also have potential for aiding glycemic control in both type 2 and type 1 diabetics [308,309]. These benefits may reflect a cGMP-mediated correction of the under-expression of glucokinase in the hepatocytes and pancreatic beta cells of diabetics [228,231,310,311]. Glucokinase functions as a “glucose sensor” in these cells to regulate insulin secretion and gluconeogenesis in a physiologically appropriate way [312,313].

## 13. Possible Limitations of this Strategy

A note of caution is in order, however. Whereas the triggers initiating the processes leading to diabetic complications are likely to have been discussed here—dicarbonyl stress, oxidative stress, an accelerated polyol pathway, and possibly increased O-GlcNAcylation (the classic mechanisms proposed by Brownlee and colleagues)—they may have downstream effects that do not readily reverse when the initiating cause is relieved [314]. Notably, changes in differentiation state may be conserved by self-reinforcing regulatory loops and altered DNA methylation patterns in the absence of the initiating stimulus [315]. Additionally, tissue fibrosis may not be readily reversed. By way of analogy, blowing out a match may do little good once the forest is ablaze. This consideration argues for diagnosing diabetes promptly and starting protective measures as soon as is feasible.

## Figures and Tables

**Table 1:** Comprehensive Control of Oxidative and Dicarbonyl Stress with Nutraceuticals as a Strategy for Prevention of Diabetic Complications.

**Inhibit NADPH oxidase activity:**
Spirulina/phycocyanin
**Promote autophagy/mitophagy/mitochondrial biogenesis**:
Sirt1 activators—Ferulic Acid, Melatonin, N1-Methylnicotinamide, Nicotinamide Riboside, Urolithin A, Thymoquinone, PQQ, Berberine, Spermidine, Astaxanthin, Nrf2 activators (see below)
**Induce mitochondrial uncoupling proteins**:
Astaxanthin, Capsaicin
**Protect the inner mitochondrial membrane with lipid-soluble scavenging antioxidants**:
Astaxanthin
**Re-couple uncoupled eNOS**:
Citrulline, High-Dose Folate
**Boost expression of antioxidant enzymes, glyoxalase 1 and glutathione**:
Lipoic Acid; Sulforaphane; Melatonin; Glycine; Zinc; H_2_S generators: Taurine, N-Acetylcysteine
**Support glutathione synthesis**:
N-Acetylcysteine; Glycine
**Promote NADPH generation via the pentose phosphate pathway**:
High-dose Thiamine/Benfotiamine; Nrf2 activators (see above)

**Table 2:** Supplementation Program for Diabetics.

***Powder or Bar***
Per serving:
Spirulina—7.5 g
Citrulline—2 g
Glycine—5 g
Taurine—1 g
*2 servings daily*
***Capsules***
4 caps provide:
Ferulic Acid—250 mg
Nicotinamide Riboside—250 mg
Lipoic Acid—600 mg
N-Acetylcysteine—600 mg
Berberine—500 mg
Astaxanthin—12 mg
Capsaicin (as cayenne pepper)—3 mg
Spermidine—10 mg
PQQ—10 mg
Folate—20 mg
*4 caps twice daily*
***Tablet—Insurance Formula***
Essential vitamins and minerals—including Zinc—25 mg, Thiamine—50 mg
1 tablet twice daily
***Melatonin Cap***—5 mg at bedtime
